# Custom healing abutment performing The Spider Web Technique. A series of two cases

**DOI:** 10.4317/jced.63239

**Published:** 2025-10-17

**Authors:** Carlos Polis-Yanes, Carla Cadenas-Sebastián, Sonia Egido-Moreno, José López-López

**Affiliations:** 1 DDS, Associate professor. Department of Odontoestomatology. Faculty of Medicine and Health Sciences, University of Barcelona, University Campus of Bellvitge, L’Hospitalet de Llobregat, Barcelona, Spain; 2 MD, Full professor. Department of Odontoestomatology. Faculty of Medicine and Health Sciences, University of Barcelona, University Campus of Bellvitge, L’Hospitalet de Llobregat, Barcelona, Spain. Oral Health and Masticatory System Group (Bellvitge Biomedical Research Institute) IDIBELL, University of Barcelona, L’Hospitalet de Llobregat, Barcelona, Spain

## Abstract

**Background:**

Bone and soft tissue remodeling after tooth loss is an inevitable physiological process. By placing immediate dental implants and performing a custom healing abutment (CHA), we can help achieve a stable emergence profile and reduce tissue volume loss. Purpose: The purpose of this work is to present a series of two clinical cases to show the Spider Web Technique for the creation of custom healing abutments (CHAs) chair-side in immediate implants.

**Material and Methods:**

We present two cases of lower molars with CHAs performed chair-side in the clinic using titanium abutments and flowable composite with the Spider Web Technique as an alternative to prefabricated titanium or polyetheretherketone (PEEK) ones. A brief discussion was held providing other techniques for carrying out CHAs.

**Results:**

The creation of CHAs was simple and fast and the postoperative period was very good. After the osseointegration period, the appearance of the peri-implant tissues, as well as the bone level, were optimal for making the definitive crown.

**Conclusions:**

The Spider Web Technique provides an effective way to create a CHA around a provisional titanium abutment immediately intraoperatively chair-side with low cost and predictable results. Comparative protocolized studies with others are necessary to obtain clear conclusions and protocols.

## Introduction

The single implant currently represents an efficient and predictable way to replace a lost tooth. Current literature supports the placement of immediate implants in posterior areas as a predictable treatment (1). Sometimes it is a challenge for the implantologist to maintain the peri-implant tissues after tooth extraction, especially when immediate loading is not an option. This is an important fact given that the thickness of the soft tissue is a key factor for the stability of the level. of peri-implant marginal bone (2).

Provisionalization, when performed appropriately, has been shown to be effective in maintaining peri-implant gingival architecture (3). The creation of customized healing abutments is a clinically and scientifically proven technique to preserve gingival anatomy after the placement of an immediate implant (4).

A custom healing abutment is a personalized component created to facilitate healing of the tissues around an immediate dental implant. This abutment specifically adapts to the shape and size of the patient's socket, which helps promote better integration of the implant and maintain aesthetics during the healing process.

## Case Report

We present two clinical cases in which a CHA was created in a lower first molar (3.6 and 4.6) after placing an immediate post-extraction implant. In both cases we performed the Spider Web Technique using a titanium temporary abutment and flowable dental composite ultra polished.

Case 1

Molar 3.6 with a diagnosis of non-restorable caries with furcation involvement. Atraumatic extraction was performed with odontosection, immediate placement of a 4.8x10mm Phibo Aurea Evo ® implant in the fresh socket. After obtaining adequate primary stability, we made a CHA using a temporary titanium abutment and flowable composite (SDI Luna Flow®), creating a spider web-shaped framework to delimit the socket. Subsequently, the anatomy of the emergence profile considered most appropriate for this case was completed (following the esthetic biological contour (EBC) concept) and polished extraorally. The roots gap was filled with bone xenograft (Ti-Oss®). Antibiotic prophylaxis was administered with amoxicillin 750 mg three times a day for one week.

**Figure 1:**
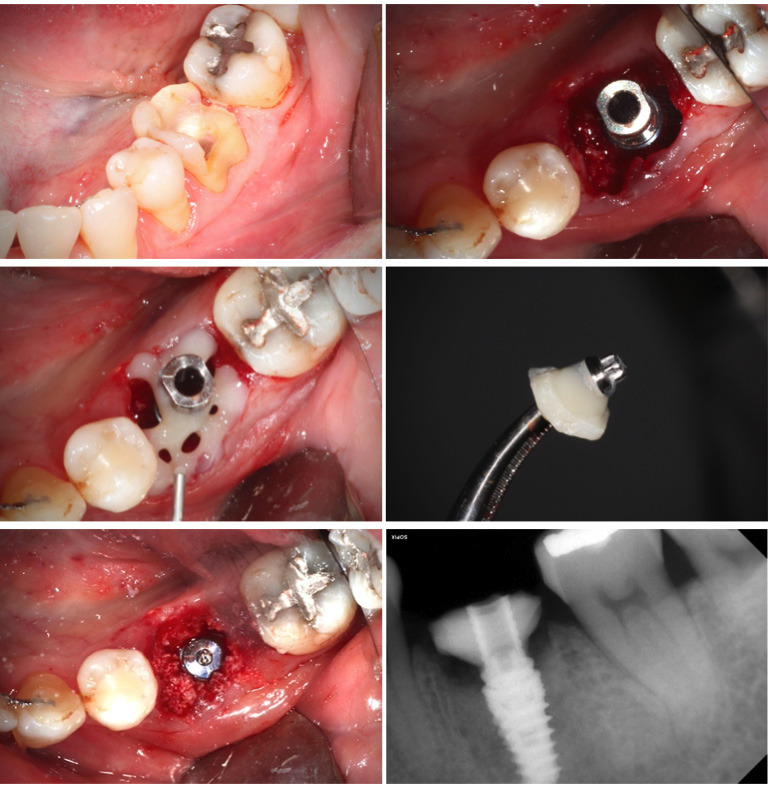
Molar 3.6 non-restorable. Implant placed. Performing spider web with Titanium abutment and flowable composite. Custom healing abutment finished and polished. Gap Filled with xenograft. Immediate x-ray.

**Figure 2:**
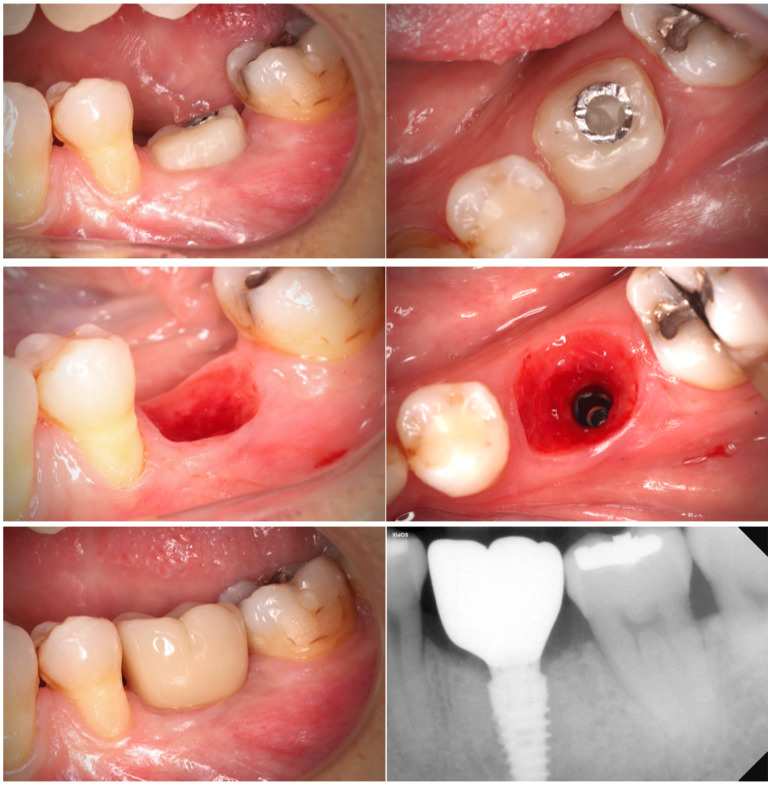
Healing after 4 months (lateral and occlusal view). Emergence profile without CHA (lateral and occlusal view). Final zirconia crown. Final x-ray.

After four months, the tissue maintenance was adequate and we made the final crown in monolithic Zr with Tibase with full digital protocol, (Figs. 1,2).

1

2

Case 2

Molar 4.6 with vertical fracture and old silver amalgam restoration. The same protocol was carried out as in case 1: extraction with odontosection, placement of a 4.8x10mm Phibo Aurea Evo ® implant in the interradicular septum, filling of the gap with xenograft (Ti-Oss®) and placement of a CHA performing our Spider Web Technique with the same process of Case 1. Antibiotic prophylaxis was administered with amoxicillin 750 mg three times a day for one week.

**Figure 3:**
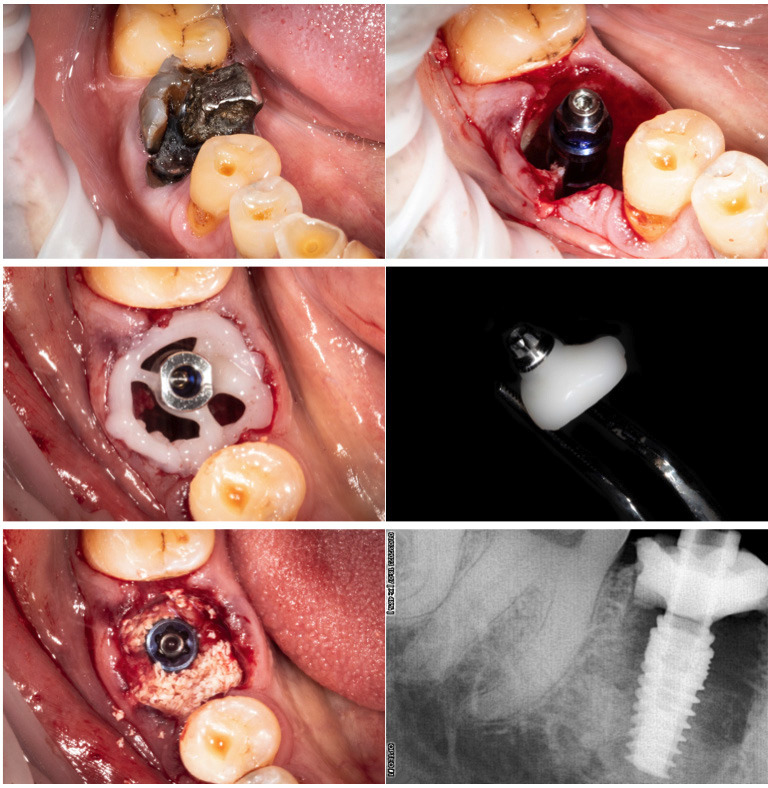
Molar 4.6 non-restorable. Implant placed. Performing spider web with Titanium abutment and flowable composite. Custom healing abutment finished and polished. Gap filled with xenograft. Immediate x-ray.

**Figure 4:**
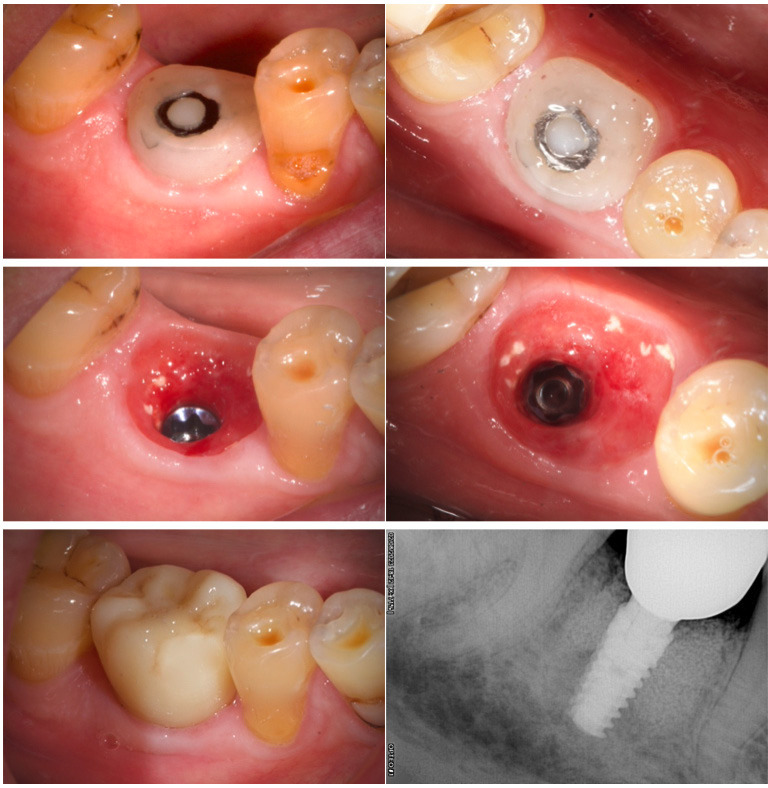
Healing after 4 months (lateral and occlusal view). Emergence profile without CHA (latteral and occlusal view). Final zirconia crown. Final x-ray.

After four months, the maintenance of the gingival anatomy was adequate and we made the final crown in monolithic Zr with Tibase with full digital protocol, (Figs. 3,4).

3

4

## Discussion

Although it is impossible to prevent tissue changes from occurring after tooth extraction, CHAs are a proven way to maintain the emergence morphology of implant-supported crowns (5). Custom healing abutments have been introduced into implantology to preserve or create natural-looking hard and soft tissue around the implant. This procedure eliminates the second stage and reduces the time elapsed from the manufacture of the final prostheses. Among the materials described, PEEK, PMMA, zirconia, resin composite, and titanium have been used (4). Lertwongpaisan et al. (2023) propose the use of digitally manufactured titanium CHA prior to tooth extraction and obtaining successful results (5). Chokaree et al. (2024) compare the use of non-customized titanium healing abutments with customized CHA, made of prefabricated polyetheretherketone (PEEK) with digital impressions. The morphology of the customized healing abutment demonstrated a better trend in preservation of peri-implant soft tissue, esthetic outcomes, and lower patient discomfort in immediate implant sites (6). Lops et al. (2023) in a systematic review and meta-analysis on CHAs carried out using CAD/CAM in the aesthetic area concluded that they do not represent a real advantage over prefabricated ones and also represent an increase in cost and time; and they warn of the importance of correctly assessing in which cases to use them (7). Alalawi (2024). proposes the creation of CHAs making a 1-piece computer-aided design and computer-aided manufacturing (CAD-CAM) with resin (8). Gomez-Meda et al. (2021) explain the importance of obtaining an adequate emergency profile for implants in order to obtain predictable aesthetic results and tissue stability around the implant. And they show how to design esthetic biological contour (EBC) concept (9).

## Conclusions

The Spider Web Technique provides an effective way to create a CHA around a provisional titanium abutment immediately intraoperatively chair-side with low cost and predictable results. Comparative protocolized studies with others are necessary to obtain clear conclusions and protocols.

## Data Availability

The datasets used and/or analyzed during the current study are available from the corresponding author.
